# Flow through a circular tube with a permeable Navier slip boundary

**DOI:** 10.1186/1556-276X-6-389

**Published:** 2011-05-17

**Authors:** Barry James Cox, James Murray Hill

**Affiliations:** 1 Nanomechanics Group, School of Mathematical Sciences, University of Adelaide, SA 5005, Australia

## Abstract

For Newtonian fluid flow in a right circular tube, with a linear Navier slip boundary, we show that a second flow field arises which is different to conventional Poiseuille flow in the sense that the corresponding pressure is quadratic in its dependence on the length along the tube, rather than a linear dependence which applies for conventional Poiseuille flow. However, assuming that the quadratic pressure is determined, say from known experimental data, then the new solution only exists for a precisely prescribed permeability along the boundary. While this cannot occur for conventional pipe flow, for fluid flow through carbon nanotubes embedded in a porous matrix, it may well be an entirely realistic possibility, and could well explain some of the high flow rates which have been reported in the literature. Alternatively, if the radial boundary flow is prescribed, then the new flow field exists only for a given quadratic pressure. Our primary purpose here is to demonstrate the existence of a new pipe flow field for a permeable Navier slip boundary and to present a numerical solution and two approximate analytical solutions. The maximum flow rate possible for the new solution is precisely twice that for the conventional Poiseuille flow, which occurs for constant inward directed flow across the boundary.

## Introduction

A body of evidence currently exists in the literature [1,2] that suggests that fluid flow rates in carbon nanotubes are considerably in excess of that predicted by the conventional Poiseuille flow field, even taking into account a slip boundary condition. Some of this evidence has been re-appraised and certain errors in experimental measurements have been strongly suspected [3]. Despite such findings, there is also a body of independent evidence to suggest that individual molecules (say of water) may achieve flow velocities in carbon nanotubes *in vacuo *as high as 1,000 m/s [4]. As noted in [4] these high velocities are greatly reduced if allowance if made for non-vacuum effects. On balance there is sufficient evidence to suggest that fluid flow through carbon nanotubes may be quite different to conventional Poiseuille flow. In this article we ask, under what conditions might other flow fields become available for Newtonian viscous flow in a tube subject to a linear Navier slip boundary condition? At the nanoscale the continuum hypothesis does not apply and the particular point of view adopted in this article is that the Navier-Stokes equations are the best available approximation to nanoscale fluid flow, which together with the linear Navier slip boundary condition might reflect certain nanoscale effects. It is widely believed that confined flows at the nanoscale exhibit both density and viscosity variations closer to the boundaries [5] which arise in part from molecular van der Waals interactions with the boundaries. In this situation the basic governing flow equations would need to be modified to incorporate the density and viscosity variations. However, even with such modifications, one would still expect comparable formal mathematical solutions to those arising from the case of constant density and viscosity as outlined below.

Conventional Poiseuille flow [6,7] arises from a pressure which is linear in the dependence along the length of the tube. We find that an exact flow arises satisfying a linear Navier slip boundary condition and arising from a pressure which is quadratic in the dependence along the length of the tube. However, quite remarkably, this second new flow field only exists for a prescribed permeability on the boundary. That is, assuming that the pressure is determined from experimental data, the radial flow velocity at the boundary must be prescribed quite precisely to achieve the quadratic pressure flow field. In the context of carbon nanotubes embedded in a matrix, the boundary may well be permeable at the molecular level, either naturally or arising from defects. Alternatively, if the radial boundary flow is prescribed, then the new flow field exists only for a given quadratic pressure. In other words, both the radial boundary flow and the quadratic pressure cannot be prescribed simultaneously. At present there is insufficient evidence in the literature to conclude whether or not carbon nanotubes have permeable boundaries. Our purpose here is to report that an exact solution of the Navier boundary layer equations with a linear Navier slip boundary can be determined which is different from conventional Poiseuille flow and corresponds to a quadratic pressure and a prescribed permeability on the boundary.

First we comment that there exists in the literature a number of solutions of the Navier-Stokes equations relating to laminar flow in both porous rectangular channels (see for example Berman [8] and Yuan [9]) and porous cylindrical pipes (see for example White [10], Terrill and Thomas [11] and Terrill [12,13]), and there are additional references cited in these articles. Second, assuming axially symmetric flow, it transpires that since the radial fluid velocity *u*(*r,z*) is assumed to be a function of the radial coordinate *r *only, namely *u*(*r*), then the full Navier-Stokes equations happen to reduce to boundary layer flow in a cylindrical pipe. Accordingly, in the problem studied here, we do not need to assume boundary layer flow but, as described in the following section, the final governing equations are identical to those arising from the boundary layer approximation. This observation is consistent with the observation of Burde [14], where it is noted that certain solutions of the boundary layer equations for axially symmetric pipe flow are also exact solutions of the full axially symmetric Navier-Stokes equations. Finally, we also comment that for fluid flow at the nanoscale, it is generally believed that there are insufficient numbers of molecules for the Newtonian fluid flow equations to apply. However, any as yet unformulated alternative theory would necessarily be probabilistic in nature and no doubt far more complicated. Again we emphasise that the approach adopted here is to recognise that at present the Newtonian fluid flow equations offer the best approximation, and that the use of the Navier slip boundary condition might well be sufficient to reflect certain aspects of nanoscale fluid flow behaviour.

In the following section we present the Navier-Stokes governing equations for axially symmetric Newtonian flow in a tube, and we determine two coupled non-linear ordinary differential equations arising from incompressible flow with radial velocity *u *as a function of the radius *r *only. In the section thereafter we detail results arising from a full numerical solution and compare them with two analytical approximate solutions. Details of the approximate analytical solutions are presented in Appendix A and B. Some brief conclusions are presented in the final section of the article.

## Governing equations

**Figure 1:**
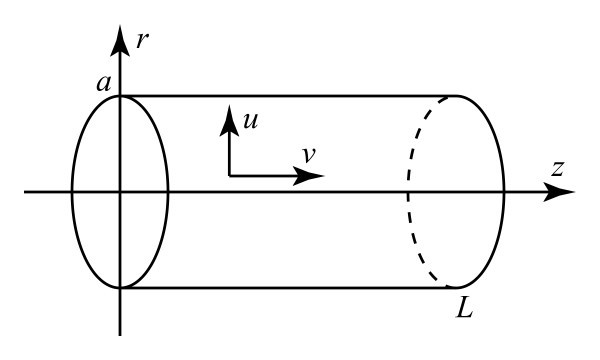
**Flow in a cylinder of radius *a *and length *L*, with radial velocity *u *and axial velocity *v***.

We consider axially symmetric incompressible flow of a Newtonian fluid in a nanotube, with a linear Navier slip boundary condition applying on the tube wall. In cylindrical polar coordinates (*r*, *θ*, *z*) with radial velocity *u*(*r*, *z*) and axial velocity *v*(*r*, *z*) as illustrated in Figure 1, the three basic partial differential equations for axially symmetric flow arising from the Navier-Stokes equations and the condition of incompressibility are(1)(2)(3)

where *μ *is the viscosity, *ρ *is the density and *p *is the pressure function. Further, ▽^2 ^denotes the usual axially symmetric Laplacian which is defined by ▽^2^=∂^2^/∂*r*^2^+(1/*r*)∂/∂*r*+∂^2^/∂z^2 ^Assuming a constant flow *u*_0_, through the tube wall and a linear Navier slip boundary condition, the two boundary conditions at the wall, *r *= *a*, become(4)

where ℓ denotes the slip-length and noting that we need the positive value of the derivative ∂*v*/∂*r *at the boundary. We comment that a full discussion relating to the origins and history of the no slip boundary condition in fluid mechanics and of the validity of the Navier slip boundary condition in connection with macroscopic fluid mechanics is presented in an informative appendix in Goldstein [15]. However, at the nanoscale the no slip boundary condition is thought not to apply and a slip boundary is believed to be far more realistic [16,17]. In addition, along the tube axis we require(5)

Now with the assumption *u *= *u*(*r*), the condition of incompressibility gives *v *= *v*_1_(*r*)*z *+ *v*_0_(*r*), where *v*_0_(*r*) denotes an arbitrary function of *r *and(6)

and the first Navier-Stokes equation simplifies to become(7)

This equation can be trivially integrated to yield(8)

where *P*(*z*) denotes an arbitrary function of *z*. On substitution of this expression for the pressure function into the second Navier-Stokes equation we may deduce a system which is formally identical to that arising from the standard boundary layer approximation for axially symmetric pipe flow, namely(9)

However, we emphasise that this equivalence hinges on the assumption *u*(*r*,*z*) = *u*(*r*), but as previously noted, Burde [14] gives other examples of solutions of the boundary layer equations which are also exact solutions of the full Navier-Stokes equations.

In the usual way we introduce a stream-function *ψ *(*r*, *z*) such that(10)

and we assume that(11)

for certain functions *f *(*r*) and *g*(*r*). From these equations we have(12)

where the primes denote differentiation with respect to *r*. If we now set(13)

where *ξ *= *a*^2 ^- *r*^2^, then we have(14)

where now primes denote differentiation with respect to *ξ*. From these relations we obtain(15)

from which we deduce that(16)

for certain constants *C*_1 _and *C*_2_, and that(17)(18)

where *v *= *μ*/*ρ *denotes the kinematic coefficient of viscosity. On introducing new variables(19)

we may show that these equations become(20)(21)

where the new constants *α *and *β *are defined by(22)

and the above ordinary differential equations must be solved subject to the boundary conditions(23)

where *A*_0 _= *au*_0_/2*v *and *γ *= 2ℓ/*a *and in this context primes denote differentiation with respect to *x*. Also we are assuming that *A*"(0)>0 and *B*"(0)>0 and have imposed a condition on *B*(*x*), such that *B*(1) = 0. Now since(24)

must remain finite along the axis *r *= 0, we have the additional requirements that both *A'*(1) and *B' *(1) remain finite. We comment that Terrill and Thomas [11] provide a comprehensive account of asymptotic and numerical solutions of the single equation (20) for the case of no slip. White [10] also provides a power series solution of the same problem but again for the case of no slip. As far as the authors are aware, to date an analysis for both *A*(*x*) and *B*(*x*) non-zero and for the case of slip has not appeared in the literature.

In the case *A*(*x*) ≡ 0, we have(25)

which integrates to yield(26)

where *D*_1 _is an arbitrary constant. We require *B" *to remain finite at *x *= 1 and therefore *D*_1 _= 0. A further integration gives(27)

where *D*_2 _is another arbitrary constant. From *B*'(0) = *γB*"(0) we may deduce the relation(28)

and the velocity field becomes *u*(*r*,*z*) ≡ 0 and(29)

which is the standard equation for fully developed laminar flow with a slip length ℓ.

To calculate the flow rate *Q *we integrate the axial velocity over the cross-sectional surface of the tube at *z *= *L*. The angular integral is trivial and gives(30)

Substituting *x *= 1-*r*^2^/*a*^2^, and *v*(*r*,*z*) = 4*v*[*zA*'(*x*)+*B*'(*x*)]/*a*^2^, gives(31)

and using the boundary conditions that *A*(1) = *B*(1) = 0 gives(32)

## Numerical results

In this section we illustrate the general features of the flow using both numerical and approximate analytical solutions. We find that for general values of the parameters *α*, *β *and *γ*, the solution only exists for a particular value of the permeability *A*_0_. This is also a feature of the approximate solutions which are given in the appendices. Also we show that the usual boundary value problem with a specific value of *A*_0 _will generally not converge and only a precise value of *A*_0 _enables the general numerical boundary value solution to be obtained. For these reasons we adopt the following procedure to find our numerical solution.

We first begin with the Bessel function approximate solution as outlined in Appendix A. Since the analytical solution has a logarithmic singularity at *x *= 1 one of the arbitrary constants is immediately forced to zero and therefore only two boundary conditions are necessary to fully determine the solution of the third-order equation. However, the full numerical solution of the non-linear third-order ordinary differential equation (20) requires three boundary conditions and therefore we provide the boundary condition *A' *(1) = *A*_1 _as determined from the approximate solution and then supply the approximate solution to the numerical solver which then converges to the required solution. A similar issue exists to determine the solution for *B*(*x*) and again the approximate analytical solution is used to provide the boundary condition *B' *(1) = *B*_1 _for the numerical solution. We comment that while these boundary conditions can be thought of as artificial, the approach is justifiable since the linearisation of the governing differential equations is performed using values for *A *and its derivative at *x *= 1, and therefore one should expect that the approximate solution will be most accurate at this point.

The numerical solutions are developed in Maple using the dsolve () routine with the options: type = numeric, method = bvp[middefer], maxmesh = 256, which implements a boundary value problem solver using a midpoint scheme with deferred corrections and up to 256 discrete points. We also need to provide the approximate analytical solutions for *A*(*x*), *A' *(*x*), *B*(*x*) and *B' *(*x*), which are derived in Appendix A. For the purpose of this comparison we assume the parameters of viscosity *μ*, density *ρ*, nanotube length *L *and pressure upstream *P*_0 _and downstream *P_L_*are constant with the values as given in Table 1. We repeat the calculation using a value of nanotube radii *a *= 2 nm, and five specific values of slip length ℓ, being 0, 3, 6, 12 and 24 nm.

**Table 1:** Table of constant parameters for the analysis of water flow through a carbon nanotube

Parameter name	Symbol	Value	Units
Tube radius	*a*	2 × 10^-9^	m
Tube length	*L*	100 × 10^-9^	m
Dynamic viscosity	*μ*	10^-3^	Pa s
Density	*ρ*	10^3^	kg m^-3^
Kinematic viscosity	*v*	10^-6^	m^2^s^-1^
Pressure upstream	*P*_0_	10^5^	Pa
Pressure downstream	*P_L_*	0	Pa

The degree to which the pressure profile in the nanotube resembles a linear profile or a quadratic profile is measured by the introduction of a new non-dimensional parameter *ε *which measures the relative magnitude of *C*_1 _and *C*_2_; namely we define *ε *by the relationship(33)

which is such that for *ε *= 0 there is no flow through the wall and *ε *= ∞ there is no flow through the tube entrance. Thus, *ε *provides a measure of the relative amount of fluid flow through the permeable boundary. By solving the pressure relationship (16) subject to the boundary conditions at *z *= 0 and *z *= *L *and the relative sizes of of *C*_1 _and *C*_2 _depending on *ε *we obtain(34)

where Δ*P *= *P*_0_-*P_L_*. We comment that when *ε *= 0 then the pressure term is entirely linear, *C*_1 _= 0 and *C*_2_=Δ*P*/*ρL *and the solution is standard Poiseuille flow. As *ε *approaches infinity then *C*_1_→2Δ*P*/*ρL*^2 ^and *C*_2_→0 and the pressure profile is entirely quadratic in the sense that d*P*/d*z *= 0 at *z *= 0.

**Figure 2:**
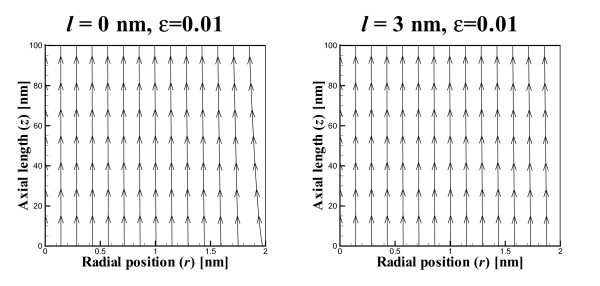
**Flow field showing streamlines for *ε *= 0.01 and slip length ℓ ∈{0,3}nm and *u*_0_={-99, -690} nm s ^-1^**.

**Figure 3:**
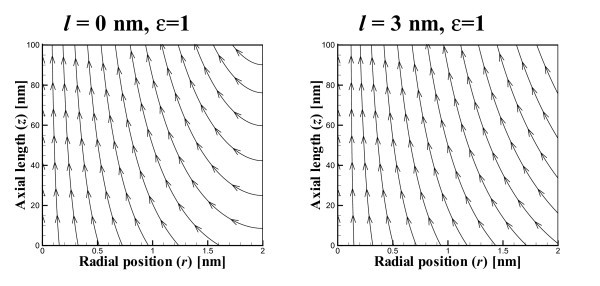
**Flow field showing streamlines for *ε *= 1 and slip length ℓ ∈{0,3} nm and *u*_0_={-5,-340} *μ*ms^-1^**.

**Figure 4:**
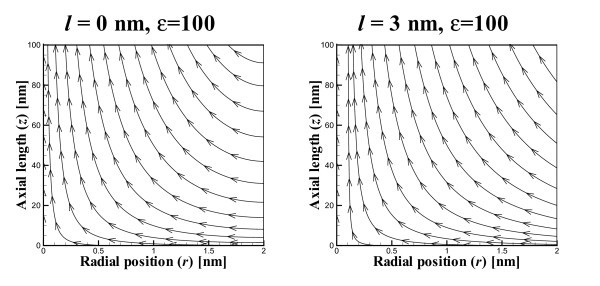
**Flow field showing streamlines for *ε *= 100 and slip length ℓ ∈{0,3} nm and *u*_0_={-9.9,-69} *μ*ms^-1^**.

In Figure 2, we show the flow field resulting from a low value of *ε *= 0.01, which corresponds to an almost linear pressure gradient in a nanotubes of radius *a *= 2 nm and length *L *= 100 nm. Corresponding graphs are displayed in Figures 3 and 4 which show the flow fields for values of *ε *= 1 and 100, respectively. The leftmost graphs (ℓ = 0) show that as expected the inflow at the tube wall is perpendicular to the tube axis when there is no slip on the tube wall boundary. It also shows that as *ε *increases, in other words as the quadratic pressure term dominates, then the outflow originates exclusively from the tube wall and the inflow at the tube opening is negligible. The corresponding graphs on the right shown in Figures 2, 3 and 4 are for a slip length of ℓ = 3 nm. In these graphs we again see that as *ε *increases and the quadratic pressure term dominates, the flow at the tube opening becomes negligible. The salient difference between the graphs on the left and those on the right is that in the rightmost graphs the flow lines at the tube wall are not perpendicular to the axis, which is a feature of the Navier slip condition at that boundary.

**Figure 5:**
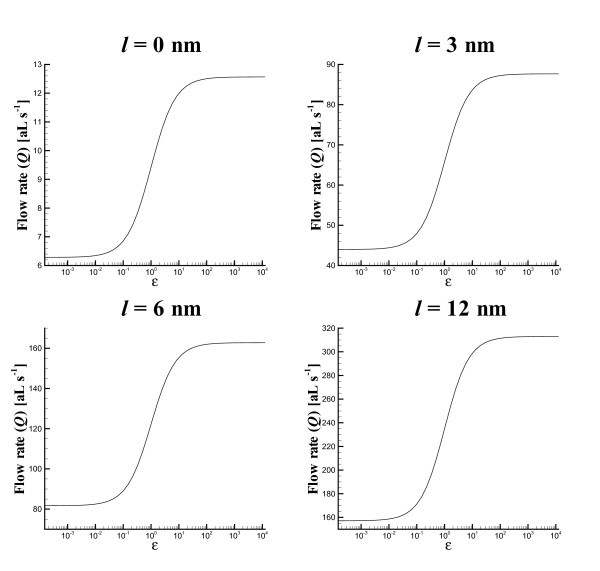
**Flow rates *Q *for tubes of radius *a *= 2 nm, length *L*= 100 nm and a slip length of ℓ ∈{0,3,6,12} nm**. Note that the units are 10^-18^Ls^-1 ^= *a *Ls^-1^.

In Figure 5, we graph the flow rate *Q *for a nanotube of radius *a *= 2 nm, length *L *= 100 nm and various slip lengths against the parameter *ε*. We note from this graph that for that most values of the slip length ℓ the ratio of flow rates for *ε *≪ 1 and *ε *≫ 1 is precisely 1.2. However, we note that for a slip length of *l *= 3 *μ*m (not graphed here), we find that this ratio begins to degrade and is approximately 1:1.72. This indicates that for larger slip lengths the inflow from the permeable nanotube wall cannot completely replace all the inflow from the open tube end at *L *= 0. We would expect that this ratio reduces even further for larger values of the tube radius a and the slip length ℓ.

**Figure 6:**
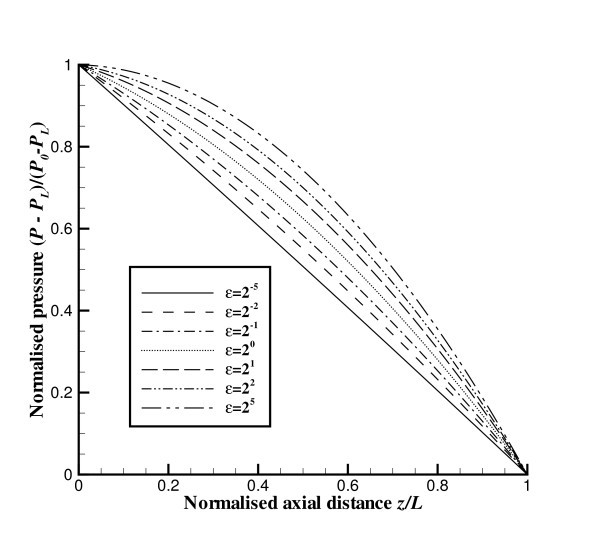
**Normalised pressure (*P *-*P_L_*)/(*P*_0 _-*P_L_*) as a function of normalised axial distance ***z/L*****. Five specific values of *ε *are shown.

In Figure 6, we graph the normalised pressure (*P *- *P_L_*)/(*P*_0 _- *P_L_*) as a function of the normalised distance along the axis *z*/*L *for various values of the parameter *ε*. We comment that for *ε *≪ 1 we obtain the expected linear relationship between pressure and distance. For *ε *≫ 1 we obtain a pressure with a quadratic dependence on distance such that the derivative of the pressure d*P*/d*z *approaches zero at *z *= 0 and at *z *= *L *the derivative of the pressure d*P*/d*z *for large *ε *approaches exactly twice the value of the linear relationship obtained for *ε *≪ 1.

## Conclusions

The problem of fluid flow through carbon nanotubes is believed to involve flow rates which are well in excess of conventional Poiseuille pipe flow. Here we have determined a new exact pipe flow from the Navier-Stokes equations which only becomes available for a certain prescribed flow through an assumed permeable boundary. While such a flow field is non-physical for conventional pipe flow, for a carbon nanotube embedded in a porous matrix, the higher than normal flow rates may well be due to additional permeable boundary flow. Simply not enough is known regarding flow in nanotubes and this possibility may be physically realistic. However, the new flow field gives rise to enhanced flow rates which are at most twice the conventional Poiseuille flow rates and occur for an injected radial flow velocity across the permeable boundary, but certainly does not explain some of the extraordinarily high flow rates that have been reported in the literature for carbon nanotubes.

## Competing interests

The authors declare that they have no competing interests.

## Authors' contributions

Both authors were involved in the conception of the study, developing the mathematical model, performing the analysis and drafting the manuscript. Both authors read and approved the final manuscript.

## Appendix

### A Bessel function approximate solution

In order to determine an approximate solution to (20) we linearise the equation by approximating *A*(*x*) and *A' *(*x*) occurring in the non-linear terms by specific values of these functions at a particular point. To this end we use the values *A*(1) = 0 and *A' *(1) = *A*_1_, which yields the modified linear equation(35)

which is a second-order linear differential equation in *A*' and which has the general solution(36)

where *J_v_*(z) and *Y_v_*(*z*) denote the usual Bessel functions of the first and second kinds, respectively, and *D*_1 _and *D*_2 _are arbitrary constants which must be determined. We now apply the boundary condition that *A' *(1) = *A*_1_, and we may determine that *D*_1 _= *A*_1 _+ *α*/*A*_1 _and *D*_2 _= 0, giving(37)

and upon integrating and applying the boundary condition *A*(1) = 0 we obtain(38)

We now determine the value of *A*_1 _from the Navier slip boundary condition (23)_3 _at *x *= 0 which gives(39)

which is a transcendental equation that may be solved numerically to determine the value of *A*_1_. With the constant *A*_1 _so determined, the problem is over determined in the sense that the tube permeability *A*_0_, must be prescribed and also determined from (38) by substituting *x *= 0. The interpretation of this result is that the new flow field found here is only physically meaningful for a precise combination of the three variable, the change in pressure, the slip length and the tube permeability, namely *α*,*γ *and *A*_0_. This result is also supported by the numerical analysis where we find that a convergent numerical solution is only available for a specific value of *A*_0_, which lies within a limited range.

To develop a corresponding approximate solution for (21) we adopt the same method as above and assume that the differential equation can be approximated with the fixed values of *A*(*x*) and *A' *(*x*) at *x *= 1. This allows us to write(40)

where, as before, *A*_1 _= *A' *(1). This is an ordinary linear differential equation which is second-order for *B*' and has the solution(41)

where *J_v_*(z) and *Y_v_*(*z*) are Bessel functions and *D*_3 _and *D*_4 _are arbitrary constants. We require that the flow velocity be well defined at *x *= 1 and therefore the constant *D*_4 _= 0, and on applying the Navier slip boundary condition gives(42)

and therefore(43)

and substituting this value for *D*_3 _into (41) and integrating gives the solution for *B*(*x*). On imposing the boundary condition that *B*(1) = 0 we obtain(44)

### B Log sine function approximate solution

We include a second approximate solution in this appendix, since again it may be formally solved and it reinforces the fact that the new solution only exists for a specific value of the radial velocity at the tube wall. In addition to the approximate solution of Appendix A we may also derive an independent approximate solution by considering the behaviour of (20) in the region very close to the singular point *x *= 1. In this case we choose to neglect the term (1-*x*)*A*"' and since *A*(1) = 0 we also suppress the *AA″ *term which leaves(45)

which is a non-linear but separable first-order equation in *A' *(*x*). Solving and applying the boundary conditions *A*(0) = *A*_0 _and *A*(1) = 0 we obtain the solution(46)

where log(*z*) denotes the natural logarithm. Now applying the Navier slip boundary condition (23)_3 _at *x *= 0 we obtain the relationship(47)

where  and this equation is quadratic in exp(- *A*_0_) and therefore(48)

We note that again we have an equation for *A*_0 _in terms of *α *and *γ *and therefore the system may be considered to be over determined such that the solution only exists for a precise combination of these parameters.
